# Hyperferritinemia and Hyperuricemia May Be Associated with Liver Function Abnormality in Obese Adolescents

**DOI:** 10.1371/journal.pone.0048645

**Published:** 2012-10-31

**Authors:** Solomon Chih Cheng Chen, Ya Fang Huang, Jung Der Wang

**Affiliations:** 1 Department of Pediatrics, Chia-Yi Christian Hospital, Chia-Yi City, Taiwan; 2 Department of Pediatrics, Medical College, Taipei Medical University, Taipei City, Taiwan; 3 Department of Clinical Laboratory, Pingtung Christian Hospital, Pingtung City, Taiwan; 4 Department of Public Health, National Cheng Kung University College of Medicine and Hospital, Tainan, Taiwan; Gentofte University Hospital, Denmark

## Abstract

**Background:**

The iron status in human body and its association with liver function in adolescents was rarely studied. The objective was to investigate the association among the levels of serum ferritin, uric acid and alanine aminotransferase (ALT) in adolescents.

**Methods and Results:**

A total of 2090 adolescents negative for hepatitis B surface antigen from one junior high school (786, 12–13 years), three senior high schools (973, 15–16 years) and one college (331, 18–19 years) participated in this survey. Anthropometric and biochemical measurements, including complete blood count, ALT, serum ferritin and uric acid were performed. An ALT>42 U/L was defined as elevated, a ferritin level >200 µg/L was defined as hyperferritinemia. A uric acid level >460 µmol/L in males and >340 µmol/L in females was defined as hyperuricemia. The chi-squared test, linear regression and multivariate logistic regression were used for the data analysis. Elevated ALT levels were detected in 76 (3.6%) students and were more prevalent in males than females (6.4% vs. 2.0%, p<0.001). The univariate analysis found gender, age group, body mass index, ferritin level, uric acid level and white blood cell count all to be significantly associated with elevated ALT. Linear regression showed a positive correlation among log(ferritin), uric acid level and ALT level. Elevated ALT occurred more frequently at ferritin level >100 µg/L. The logistic regression analysis found that body mass index, hyperferritinemia and hyperuricemia were significant factors associated with the ALT elevation, but gender, age, and white blood cell count were not.

**Conclusions:**

Hyperferritinemia and hyperuricemia are two independently significant factors associated with ALT elevation among obese adolescents. More studies are needed to corroborate any hypothesis related to these phenomena.

## Introduction

A higher hemoglobin level was found to be associated with elevated alanine aminotransferase (ALT) levels among healthy adolescents after controlling for gender, body mass index (BMI) and other confounding factors in our previous study [1]. We thought this association may be due to elevated levels of iron because iron is the major metal carried by hemoglobin in the human body. Another study has also reported a positive association between iron stores and serum ALT levels in healthy teenagers [2].

Iron in the human body acts as a catalyst capable of inducing the production of free radicals, leading to oxidative damage to DNA, lipids and proteins [3]. Iron overload may increase free-radical production and impair hepatic mitochondrial respiration and hepatocellular calcium homeostasis [4], and it has been known as an important mediator of hepatic oxidative stress and disease progression in chronic hepatitis C virus infection [4], [5]. Oxidative stress was considered to be a key trigger in the pathogenesis of human nonalcoholic fatty liver disease (NAFLD) and cardiovascular diseases [6], [7]. Because ferritin is the major iron-storage protein in the liver where most of the extra body iron is stored, the pathogenesis of liver damage due to iron overload could be reflected by the ferritin levels [3]–[5], [8]–[10]. Some studies have shown that the histologic severity of NAFLD was proportionally related to the serum ferritin levels, and hyperferritinemia could probably serve as a predictor of advanced fibrosis in patients with NAFLD [11].

In addition to the increased ferritin levels, this present study found a significant association between the levels of uric acid and ALT, which has not yet been well explored. The uric acid level was found to be increased in most NAFLD patients [12]. It was also associated with cardiovascular diseases and metabolic syndrome in a cross-sectional population-based study [13]. Thus, the aim of this study was to investigate the association among hyperferritinemia, hyperuricemia and ALT elevation and to explore the potential risk factors for liver function abnormality among adolescents.

## Methods

### Ethics Statement

The Ethics Review Board of Pingtung Christian Hospital approved the protocol before the commencement of this study. All participants and their guardians gave signed informed consent. All data were collected for statistical analysis only, without revealing any information related to personal identification.

### Study Population

This study was based on a routine health checkup for adolescents when they entered school. A total of 2,090 adolescents negative for hepatitis B surface antigen from one junior high school (786 students, aged 12–13 years), three senior high schools (973 students, aged 15–16 years) and one college (331 students, aged 18–19 years) in Pingtung county participated in this survey in September of 2010.

### Laboratory Data and Definitions

A physical examination and blood sampling were performed after an overnight fast. Body height, body weight, complete blood counts and serum biochemistry, including alanine aminotransferase (ALT), aspartate aminotransferase, ferritin, uric acid and cholesterol levels, were measured and recorded. All biochemical analyses were performed using a Beckman Coulter LX-20 autoanalyzer (*Beckman Coulter*, Brea, CA, USA). In our hospital, an ALT level above 42 IU/L was considered to be elevated, indicating an abnormal biochemical function of the liver. The ferritin level was classified into 3 categories: ≤100, 101–200 and >200 µg/L. The ferritin level was also transformed into log(ferritin) to show its linear correlation with ALT. A ferritin level >200 µg/L was considered as hyperferritinemia. A uric acid level >460 µmol/L in males and >340 µmol/L in females was defined as hyperuricemia [14]. White blood cell (WBC) counts were analyzed on the Sysmex XE-5000 (Sysmex Corporation, Japan). SCS-1000 (Sysmex Calibrator System) was designed for calibration and verification. A WBC count >10, 000/ µL was considered as abnormal.

Hepatitis B markers, including the hepatitis B surface antigen and hepatitis B surface antibody, were measured using a radioimmunoassay (Abbott Laboratory, U.S.A.) and classified as positive or negative. Though all of the adolescents received 3 doses of the HBV vaccine during their childhood, nine participants were still found to be positive for hepatitis B surface antigen and were excluded from the analysis. The BMI was calculated in kg/m^2^ and classified into the following three categories as suggested by the Department of Health in Taiwan: normal (BMI<24 kg/m^2^), overweight (BMI≥24 but <27 kg/m^2^) and obese (BMI≥27 kg/m^2^).

### Statistical Methods

The statistical analysis was performed using SPSS 19.0 (IBM SPSS Statistics). A *P* value <0.05 was considered to represent a statistically significant difference. The chi-squared test was used to test the association between two factors for categorical data. Linear regression was used to explore the association between ALT levels and ferritin levels and between ALT levels and uric acid levels. In addition, multiple logistic regression analyses were conducted for elevated ALT levels with the following risk factors: BMI, gender, ferritin levels, uric acid levels and WBC counts. The adjusted odds ratios (ORs) were estimated and 95% confidence intervals (95% CI) were also calculated.

## Results

### Basic Factors by ALT Categories

The distribution of gender, age groups, BMI, ferritin levels, uric acid levels and WBC count in all 2,090 adolescents is summarized and stratified by the ALT categories in Table 1. An elevated ALT level was detected in 76 (3.6%) adolescents and was more prevalent in males than females (6.4% vs. 2.0%, *P*<0.001). The univariate analysis found gender, age group, BMI, ferritin level, uric acid level and WBC count all were significantly associated with elevated ALT levels. The percentage of elevated ALT increased by age group, BMI classification, ferritin level, uric acid level and WBC count (Table 1).

**Table 1:** Distribution of ALT level by gender, age group, BMI category, ferritin level, uric acid level and white blood cell count among 2,090 adolescents.

	ALT>42 IU/L(n = 76)	ALT≤42 IU/L(n = 2014)	*P* value*
Gender			
Female	27/1321 (2.0)	1294/1321 (98.0)	<0.001
Male	49/769 (6.4)	720/769 (93.6)	
Age group (years)			
12–13	15/786 (1.9)	771/786 (98.1)	<0.001
15–16	37/973 (3.8)	936/973 (96.2)	
18–19	24/331 (7.3)	307/331 (92.7)	
Body mass index			
≤24	21/1663 (1.3)	1642/1663 (98.7)	<0.001
24–27	14/226 (6.2)	212/226 (93.8)	
>27	41/201 (20.4)	160/201 (79.6)	
Ferritin (µg/L)			
≤100	25/1570 (1.6)	1545/1570 (98.4)	<0.001
101–200	31/405 (7.7)	374/405 (92.3)	
>200	20/115 (17.4)	95/115 (82.6)	
Hyperuricemia***			
No	25/1555 (1.6)	1530/1555 (98.4)	<0.001
Yes	51/535 (9.5)	484/535 (90.5)	
White blood cell count (/µL)			
≤10,000	68/1984 (3.3)	1918/1984 (96.7)	0.004**
>10,000	10/106 (9.4)	96/106 (90.6)	

Abbreviation: ALT, alanine aminotransferase; BMI, body mass index

* Chi-squared test, ** Fisher's exact test

*** A uric acid level >460 µmol/L in males and >340 µmol/L in females was defined as hyperuricemia.

### Uric acid and ferritin levels correlate with ALT levels

**Figure 1:**
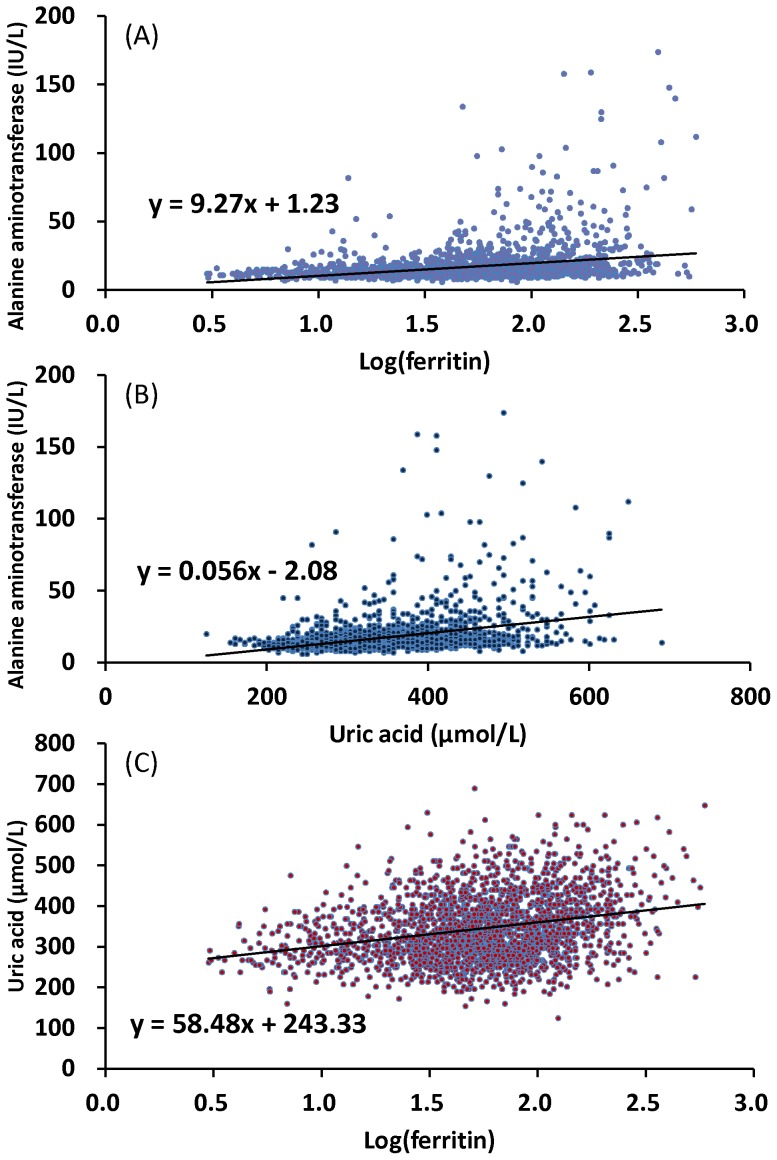
Positive correlation was found between ALT, log(ferritin) and uric acid levels among 2,090 adolescents. (A) ALT levels by log(ferritin); (B) ALT levels by uric acid levels; and (C) uric acid levels by log(ferritin).

There was a positive linear correlation between the ALT levels and log(ferritin), which was calculated with the equation y = 9.27x + 1.23 (R^2^ = 0.263, *P*<0.001) (Figure 1A). When log(ferritin) >2, i.e., a ferritin level >100 µg/L, the elevated ALT occurred more frequently. This positive correlation was also observed between the ALT levels and uric acid levels, which was calculated with the equation y = 3.35x − 2.09 (R^2^ = 0.343, *P*<0.001) (Figure 1B). Elevated levels of ALT occurred more frequently in subjects with higher uric acid levels. There was also a positive correlation between the uric acid levels and log(ferritin), which was calculated with the equation y = 0.98x + 4.09 (R^2^ = 0.272, *P*<0.001) (Figure 1C). The mean of the ferritin levels between with and no hyperuricemia groups was significantly different (88.5 µg/L vs. 72.8 µg/L, *P*<0.05).

### Ferritin levels in different groups

**Figure 2:**
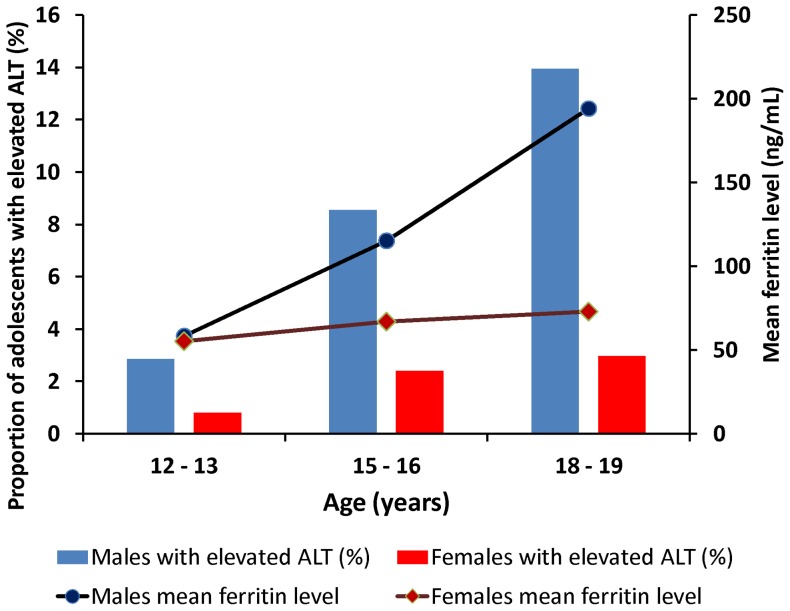
The mean ferritin levels and the proportion of adolescents with elevated ALT levels in the participants divided into three age groups.

The mean ferritin levels and the proportion of adolescents with elevated ALT levels in the three age groups of the participants are shown in Figure 2. The ferritin levels increased according to age group in the male adolescents but not in the female adolescents, and the proportion of adolescents with elevated ALT levels increased in male adolescents but not in female adolescents (Figure 2). The mean ferritin levels were significantly higher in the male adolescents than in the female adolescents (97.6 µg/L vs. 64.8 µg/L, *P*<0.001). The mean of ferritin levels at three BMI categories were 72 µg/L in BMI<24 kg/m^2^; 85 µg/L in BMI 24–27 kg/m^2^; and 107 µg/L in BMI >27 kg/m^2^, respectively (*P*<0.05).

### Risk Factors Associated with ALT Elevation

The logistic regression model to control for gender, age and WBC count resulted in the OR and 95% CI for the risk factors associated with elevated ALT in shown in Table 2. The statistically significant risk factors for elevated ALT were as follows: BMI>27, ferritin >200 µg/L, BMI 24–27, hyperuricemia and ferritin 101–200 µg/L. However, gender and age were not significant risk factors in this model.

**Table 2:** Odds ratio and 95% confidence interval (95% CI) for risk factors associated with elevated ALT among 2,090 adolescents by the logistic regression model.

	ALT (IU/L)>42/≤42	Elevated ALT (%)	Odds ratio	95% CI	*P* value
Gender					
Female	27/1294	2.0	1		
Male	49/720	6.4	2.01	0.95–4.21	0.055
Age group (years)					
12–13	15/771	1.9	1		
15–16	37/936	3.8	1.73	0.86–3.49	0.126
18–19	24/307	7.3	2.41	0.99–5.34	0.051
Body mass index					
≤24	21/1642	1.3	1		
24–27	14/212	6.2	3.81	1.85–7.85	<0.001
>27	41/160	20.4	8.74	4.64–16.47	<0.001
Ferritin (µg/L)					
≤100	25/1545	1.6	1		
101–200	31/374	7.7	2.67	1.44–4.98	0.002
>200	20/95	17.4	4.94	2.27–10.76	<0.001
Hyperuricemia*					
No	25/1530	1.6	1		
Yes	51/484	9.5	3.25	1.85–5.74	<0.001
White blood cell count (/µL)					
≤10,000	68/1918	3.4	1		
>10,000	10/96	9.4	1.17	0.51–2.70	0.709

Abbreviation: ALT, alanine aminotransferase

* A uric acid level >460 µmol/L in males and >340 µmol/L in females was defined as hyperuricemia.

**Figure 3:**
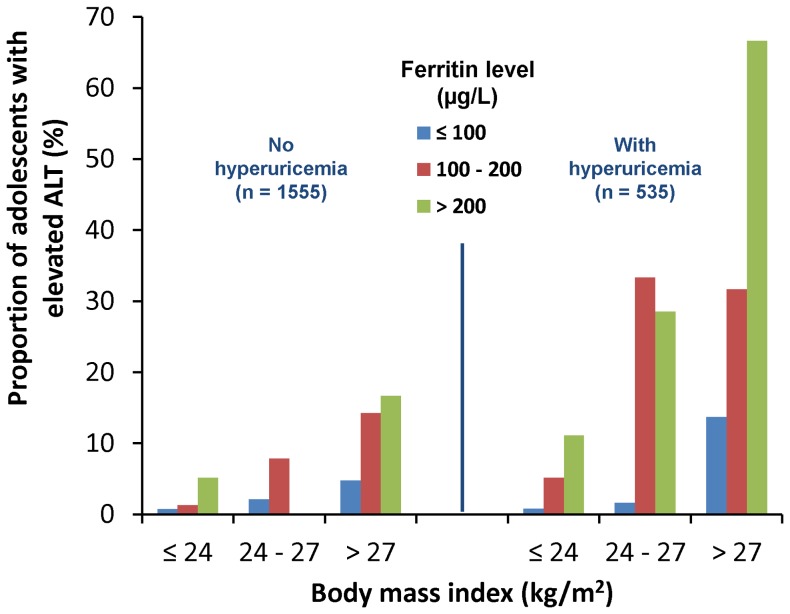
The proportion of adolescents with elevated ALT categorized by the uric acid level, body mass index and ferritin levels.

When the subjects were stratified by uric acid level, there seemed to be a dose-response relationship between the increased proportion of adolescents with elevated ALT and increased ferritin levels among the three BMI categories. This relationship was more apparent among adolescents with hyperuricemia (Figure 3).

## Discussion

Obesity is a well-known risk factor for liver function impairment. Among the adolescents included in this study, we found that subjects with a BMI>27 had the highest OR (95% CI) of 8.74 (4.64–16.47) for ALT elevation (Table 2), and the proportion of adolescents with elevated ALT also increased corresponding to the BMI level (Figure 3). In addition, this study determined that hyperferritinemia and hyperuricemia were both significantly associated with ALT elevation among adolescents after controlling for obesity, gender, age and other confounding factors. Adolescents with hyperferritinemia or hyperuricemia had a much higher proportion of elevated ALT than those without (Figure 3). There was a dose-response relationship between an increased proportion of adolescents with elevated ALT and increased ferritin levels after stratification by the three BMI categories, and this relationship was the most apparent in the group of adolescents with hyperuricemia (Figure 3). Therefore, we suggest that these three factors, including obesity, hyperferritinemia and hyperuricemia, may synergistically facilitate the ALT elevation among adolescents.

The definition of hyperferritinemia for adults was serum ferritin >200 µg/L in women and >300 µg/L in men [15]–[17]. This definition considered the gender difference in the ferritin levels and included 95% of the population within this range [17]. However, this range is quite wide and may not be consistent with clinical experience. Some studies have demonstrated that a serum ferritin >100 µg/L was associated with decreased cardiovascular fitness and increased incidences of atherosclerosis, bacterial infections, type II diabetes, cancer, gout and accelerated aging process [18], [19]. The present study corroborated previous studies and showed a rapid rise in the ALT levels if the ferritin level was greater than 100 µg/L (Figure 1A). Thus, we suggest using 100 µg/L as the upper limit of ferritin level to detect the potential harmful reaction from iron overload as early as possible.

The gender difference in the ALT elevation is a controversial issue [1], [20], [21]. The present study found that gender was significantly associated with elevated ALT levels in the univariate analysis (6.4% vs. 2.0%, *P*<0.001; Table 1) but not in the multivariate regression model after controlling for other factors (OR 2.01, 95% CI 0.95–4.21; Table 2). Thus, we concluded that gender was not a significant factor for ALT elevation. The gender difference in the ALT levels was probably due to the difference in the ferritin levels between the two genders as the mean of the ferritin levels was significantly higher in the male adolescents than in the female adolescents (97.6 µg/L vs. 64.8 µg/L, *P*<0.001). Similarly, age was significantly associated with elevated ALT levels in the univariate analysis (Table 1) but not in the multivariate regression model after controlling for other factors (Table 2). The proportion of adolescents with elevated ALT paralleled the means of the ferritin levels among the three age groups (Figure 2). The age influence on ALT elevation was also probably due to an increase in the ferritin levels. Thus, we can conclude that neither gender nor age is a significant factor for ALT elevation. Their association with ALT elevation was possibly due to the ferritin levels.

Ferritin can function as an acute phase reactant to systemic inflammatory conditions [22]–[24], and so its levels may also rise during inflammation. A recent systematic review suggested a tendency for higher ferritin level in obesity, probably due to a result of obesity-related inflammation [25]. Our finding was also consistent with this suggestion as higher ferritin levels at higher BMI categories. Because the WBC count is a common clinical marker for systemic inflammation, we used the WBC count in the multivariate model to control for the possible confounding factor of inflammation. The distribution of a WBC count >10, 000/ µL was significantly different in the two ALT groups (*P* = 0.004) but not different in the multivariate analysis (OR 1.17, 95% CI 0.51–2.70, Table 2). In addition, the study subjects were apparently healthy adolescents without any recognized inflammatory conditions. Thus, we concluded that the elevation in the ferritin levels was not due to inflammation in this study.

The present study also found a significant association between hyperuricemia and ALT elevation (Figure 1C and Table 2). Uric acid is known to be an oxidative stress marker [26] and a major antioxidant in human blood that protects against aging and oxidative stress [27]. Thus, elevated levels of uric acid could be associated with increased oxidative stress [26], [27]. Because oxidative stress is an important pathogenic marker of metabolic syndrome and cardiovascular disease, increased serum levels of uric acid might also be associated with ALT elevation [12], [27]. This study found the serum concentrations of uric acid to be positively correlated with the ALT levels (Figure 1B), and the uric acid levels were also positively correlated with log(ferritin) (Figure 1C), which is consistent with a previous report that uric acid is a cue for iron overload [28], [29].

The depletion of iron stores via phlebotomy has been shown to improve insulin sensitivity in patients with NAFLD and type II diabetes [27], [30]–[32]. Regular phlebotomy can also reduce ALT levels among chronic hepatitis C cases [33]–[38] and promote histological recovery in patients with NAFLD [31], [39]. This observation further supports the hypothesis that iron overload is relevant in the pathogenesis of NAFLD. The menstrual cycle of female adolescents is similar to a monthly phlebotomy, which can explain why the female adolescents had a much lower percentage of elevated ALT in this study (Table 1).

### Limitations

Some limitations of the present study should be mentioned. First, this was a cross-sectional study, which precluded the formation of any conclusion regarding the causal relationship among hyperferritinemia, hyperuricemia and elevated ALT levels. Second, the number of subjects with abnormal ALT levels was relatively small because this study was based on a routine entrance health checkup for apparently healthy adolescents. Thus, some numbers were lacking in Figure 3, and the dose-response trend was not shown convincingly. Third, there was no histological diagnosis of NAFLD because we could not perform a liver biopsy in the absence of a clear clinical indication in these apparently healthy adolescents. Fourth, we did not perform ultrasonography to diagnose fatty liver or NAFLD. Instead, we used elevated ALT levels as a proxy indicator, which was common in our daily practice. Fifth, alcohol consumption was not considered in the study population because it was not a common habit among Taiwanese adolescents; thus, the confounding effect is expected to be minimal. We did not check hepatitis C because its prevalence was low among young generation in Taiwan [40], [41]. We didn't investigate hemochromatosis because it is not frequent in Taiwan, only noted in some particular diseases like hemoglobin H disease [42], chronic hepatitis B or C [43]. Sixth, the use of WBC as a proxy indicator for inflammation might be not very accurate, better indicator like C-reactive protein should be considered in future study.

## Conclusions

This study explored the positive correlation among the levels of ferritin, uric acid and ALT. Both hyperferritinemia and hyperuricemia had a significant association with liver function abnormality among these apparently healthy adolescents, especially those adolescents who are obese. Because hyperferritinemia and hyperuricemia are both clinically modifiable factors, these results could be helpful for the treatment and education of those adolescents with abnormal liver function without a definite cause. More studies are needed to corroborate any hypothesis related to these phenomena.
