# Correction: Hyperferritinemia and Hyperuricemia May Be Associated with Liver Function Abnormality in Obese Adolescents

**DOI:** 10.1371/journal.pone.0173849

**Published:** 2017-03-07

**Authors:** Solomon Chih Cheng Chen, Ya Fang Huang, Jung Der Wang

There is an error in the first author’s affiliation. The correct affiliation is: Department of Pediatrics, School of Medicine, College of Medicine, Taipei Medical University, Taipei City, Taiwan.
